# The testicular soma of *Tsc22d3* knockout mice supports spermatogenesis and germline transmission from spermatogonial stem cell lines upon transplantation

**DOI:** 10.1002/dvg.23295

**Published:** 2019-04-19

**Authors:** Hai Zhou, Zhen Zeng, Frank Koentgen, Mona Khan, Peter Mombaerts

**Affiliations:** ^1^ Max Planck Research Unit for Neurogenetics Frankfurt Germany; ^2^ Ozgene Pty Ltd Bentley Western Australia Australia

**Keywords:** germline stem cell, GILZ, spermatogenesis, SSC

## Abstract

Spermatogonial stem cells (SSCs) are adult stem cells that are slowly cycling and self‐renewing. The pool of SSCs generates very large numbers of male gametes throughout the life of the individual. SSCs can be cultured in vitro for long periods of time, and established SSC lines can be manipulated genetically. Upon transplantation into the testes of infertile mice, long‐term cultured mouse SSCs can differentiate into fertile spermatozoa, which can give rise to live offspring. Here, we show that the testicular soma of mice with a conditional knockout (conKO) in the X‐linked gene *Tsc22d3* supports spermatogenesis and germline transmission from cultured mouse SSCs upon transplantation. Infertile males were produced by crossing homozygous Tsc22d3 floxed females with homozygous ROSA26‐Cre males. We obtained 96 live offspring from six long‐term cultured SSC lines with the aid of intracytoplasmic sperm injection. We advocate the further optimization of Tsc22d3‐conKO males as recipients for testis transplantation of SSC lines.

## 1 INTRODUCTION

Spermatogonial stem cells (SSCs) are adult stem cells that continuously undergo self‐renewal to maintain the undifferentiated state, and that differentiate to form mature spermatozoa throughout the lifetime of males (Kanatsu‐Shinohara & Shinohara, 2013). Theoretically, a single SSC yields ~4,096 haploid gametes by passing through successive mitoses and one meiosis—a process that takes ~35 days in mouse. SSCs reside on the basement membrane of the seminiferous tubules in the testis. A specialized microenvironment that is termed a niche supports the self‐renewal and differentation of SSCs (Oatley & Brinster, 2012). The widely used biological assay to assess SSC activity within a population of cells entails the transplantation of SSCs by microinjection into the testes of infertile mice (Brinster & Avarbock, 1994; Brinster & Zimmermann, 1994). Transplanted cells give rise to fertile spermatozoa and then to donor cell‐derived offspring either by natural mating or with the aid of an assisted reproductive technology such as intracytoplasmic sperm cell injection (ICSI). Long‐term culture of mouse SSCs became possible by adding to the medium self‐renewal factors such as glial cell line‐derived neurotrophic factor (GDNF; Kanatsu‐Shinohara et al., 2003; Kubota, Avarbock, & Brinster, 2004). It is estimated that only a small fraction (<1%) of the cells of an SSC line harbor the stem cell potential (Kanatsu‐Shinohara & Shinohara, 2013). Genetic manipulation of the genome of SSC lines has enabled the generation of genetically modified strains of mice (Kanatsu‐Shinohara et al., 2006; Sato et al., 2015; Wu et al., 2015) and rats (Chapman et al., 2015), but has not replaced gene targeting in embryonic stem cells.

There are two commonly used types of infertile recipients for SSC transplantation in mice. The first type is the Kit^W^/Kit^Wv^ mutant mouse, which has smaller testes that contain almost no germ cells and are devoid of spermatogenesis. But because Kit^W^/Kit^W^ homozygous mice die after birth, compound heterozygous Kit^W^/Kit^Wv^ males must be generated from crosses of mice carrying the Kit^W^ or Kit^Wv^ alleles in the heterozygous state. The second type is obtained by intraperitoneal injection of busulfan, an alkylating chemotherapeutic agent that preferentially kills spermatogonial stem cells. But the typical dose of 40 mg busulfan/kg body weight results not only in infertility, but also in substantial morbidity and mortality. In the few studies that report mortality rates, these ranged from 30% (Ma, Wang, Gao, & Jia, 2018), 31.6% (Qin et al., 2016), 60% (Ganguli et al., 2016), to as high as 87% (Wang, Zhou, Yuan, & Zheng, 2010) for 40 mg busulfan/kg body weight. In certain studies, busulfan‐treated mice received a bone marrow transplant to relieve hematopoietic suppression (Aoshima, Baba, Makino, & Okada, 2013; Kanatsu‐Shinohara et al., 2002, 2003; Ogawa, Dobrinski, Avarbock, & Brinster, 1999). We believe that, in the interest of animal welfare and the 3R principle of humane experimental technique with animals (Russell & Burch, 1959), it is imperative to explore alternative candidates for SSC transplantation recipients as they become available through new discoveries or new technological developments.

A novel candidate recipient that has emerged recently but has not been explored yet is the genetically infertile male mouse that is the centerpiece of the goGermline technology (Koentgen et al., 2016). This technology was originally developed to produce chimeric mice that give 100% germline transmission of the embryonic stem cell‐derived genome upon microinjection of embryonic stem cells into blastocysts or eight‐cell embryos. Infertile males are produced by crossing two strains that can be maintained in the homozygous and hemizygous state and that are healthy and fertile: females homozygous for a Tsc22d3 conditional (floxed) gene‐targeted mutation are crossed with males homozygous for a ROSA26‐Cre gene‐targeted mutation. There is no mortality at any stage; there is no genotyping of any mouse needed; and 100% of male offspring of the cross are Tsc22d3‐conKO and infertile. The goGermline technology appeared to us as promising to fulfill the reduction imperative of the 3R principle as source of an alternative, third‐type of recipient for SSC transplantation. Here, we report spermatogenesis and germline transmission of the SSC‐derived genome upon transplantation into the testes of infertile Tsc22d3‐conKO males.

## 2 MATERIALS AND METHODS

### 2.1 Mice

The CAG::mRFP1 strain is Tg(CAG‐mRFP1)1F1Hadj/J (Long, Lackan, & Hadjantonakis, 2005; The Jackson Laboratory, #5645). The UBI‐GFP strain is C57BL/6‐Tg(UBC‐GFP)30Scha/J (Schaefer, Schaefer, Kappler, Marrack, & Kedl, 2001; The Jackson Laboratory, #4353). The Tg(act‐EGFP) strain is C57BL/6‐Tg(CAG‐EGFP)131Osb/LeySopJ (Okabe, Ikawa, Kominami, Nakanishi, & Nishimune, 1997; The Jackson Laboratory, #6567). The D4/XEGFP strain is Tg(CAG‐EGFP)D4Nagy/J (Hadjantonakis, Gertsenstein, Ikawa, Okabe, & Nagy, 1998; The Jackson Laboratory, #3116). The B6‐iDTR strain is C57BL/6‐Gt(ROSA)26Sortm1(HBEGF)Awai/J (Buch et al., 2005; The Jackson Laboratory, #7900). The ROSA26‐EGFP strain is B6.Cg‐Tg(Gt(ROSA)26Sor‐EGFP)I1Able/J (Giel‐Moloney, Krause, Chen, Van Etten, & Leiter, 2007; The Jackson Laboratory, #7897). The B6‐GFP strain is C57BL/6‐Tg(CAG‐EGFP)1Osb/J (Okabe et al., 1997; The Jackson Laboratory, #3291). Mouse embryonic fibroblasts were prepared from strain Tg(DR4)1Jae/J (The Jackson Laboratory, #3208). The Tsc22d3‐flox strain (Koentgen et al., 2016) was established in embryonic stem cell line Bruce4; chimeras were crossed with ROSA26‐Flp in a C57BL/6 background; and the strain was then maintained in a C57BL/6 background with the *Tyr* ^*c*^ allele and the wild‐type *Tyr* allele segregating at the albino locus, and the *A* and *a* alleles segregating at the agouti locus. The ROSA26‐Cre strain was established in Bruce4, and mice were intercrossed for more than 20 generations with several backcrosses to C57BL/6 along the way. The Tsc22d3‐flox and ROSA26‐Cre strains are available to the research community. Mouse experiments were performed in accordance with the German Animal Welfare Act, the European Directive 2010/63/EU, and the institutional ethical and animal welfare guideline of the Max Planck Research Unit for Neurogenetics. Approval came from the *Regierungspräsidium* Darmstadt and the *Veterinäramt* of the City of Frankfurt.

### 2.2 SSC culture

The culture medium consisted of StemPro‐34 SFM with Stem Pro supplement (Kanatsu‐Shinohara, Ogonuki, et al., 2003; Wu et al., 2015; Zhang et al., 2014; Gibco, #10639–011), 25 μg/mL insulin (Sigma, #I1882), 100 μg/mL transferrin (Sigma, #T1428), 60 μM putrescine (Sigma, #P5780), 30 nM sodium selenite (Sigma, #S5261), 6 mg/mL D‐(+)‐glucose (Sigma, #G7021), 30 μg/mL pyruvic acid (Sigma, #P4562), 1 μL/mL DL‐lactic acid (Sigma, #L7900), 5 mg/mL bovine serum albumin (Calbiochem, #126609), 2 mM L‐glutamine (Millipore, #TMS‐002‐C), 100x β‐mercaptoethanol (Specialty Media, #ES‐007‐E), minimal essential medium vitamin solution (Gibco, #11120–052), 100x nonessential amino acid solution (Gibco, #11140–035), 1% penicillin/streptomycin (Specialty Media, #TMS‐AB2‐C), 0.1 mM ascorbic acid (Sigma, #A4034), 10 μg/mL d‐biotin (Sigma, #B4639), 20 ng/mL recombinant human epidermal growth factor (Gibco, #PMG8041), 10 ng/mL recombinant human basic fibroblast growth factor (PeproTech, #AF‐100‐18B‐250), 10 ng/mL recombinant human GDNF (PeproTech, #450–10‐250), 1 IU/mL Leukemia Inhibitory Factor (Millipore, #ESG1107), and 1% fetal bovine serum (HyClone, #SH30071.03). Cells were maintained at 37°C with 5% CO_2_.

### 2.3 Transplantation of SSCs

Tsc22d3‐conKO males at 4–12 weeks were used as recipient mice. Cell suspensions of 10–15 μL, which contained 2–3 × 10^5^ cells, were injected into seminiferous tubules (Ogawa, Aréchaga, Avarbock, & Brinster, 1997) using a micropipette (40–80 μm diameter tips). A Nikon SMZ25 stereofluorescence microscope was used to visualize green fluorescence in the testes. Mice were sacrificed by cervical dislocation, and testes collected and cryopreserved for immunofluorescence.

### 2.4 Immunofluorescence and imaging

SSC lines were cultured in 12‐ or 24‐well dishes. Cells were fixed in 4% paraformaldehyde at room temperature for 15 min, treated for 1 hr at room temperature with 0.5% Triton X‐100 and 5% normal donkey serum (Jackson ImmunoResearch Laboratories, #017–000‐121) diluted in phosphate buffered saline with Triton X‐100 (PBST), which consists of 1 g bovine serum albumin (Calbiochem, #126609), 5 mL 20% Triton X‐100 (Sigma, #P9416) and 100 mL 10 × PBS (Sigma, #P5493) to 900 mL ddH_2_O. Testes were fixed in 4% paraformaldehyde overnight, followed by cryoprotection in 30% sucrose, frozen in Optimal Cutting Temperature compound, and sectioned at 12 μm. Primary antibodies were diluted at 1:50–1:500 in 0.1% Triton X‐100 and 1% normal donkey serum, and samples incubated at 4 °C overnight or at 37 °C for 1 hr. After three 15‐min washes in PBST, samples were incubated for 1 hr at 37 °C in a 1:500 dilution of secondary antibody in PBST, then washed and covered with H_2_O containing nuclear stain 4′,6‐diamidino‐2‐phenylindole (DAPI, Thermo Fisher scientific, #D1306) for 5 min. Primary antibodies were MVH (abcam, #Ab13840) at 1:500 dilution, GILZ (Santa Cruz FL‐134, #sc‐33780) at 1:50, TRA98 (abcam, #ab82527) at 1:500, TRIM36 (abcam, #ab116212) at 1:200, TNP1 (abcam, #ab73135) at 1:150, SCP3 (abcam, #ab15093) at 1:150, WT1 (abcam, #ab89901) at 1:150, SOX9 (Merck Millipore, #AB5535) at 1:500, and CD146 (BioLegend, #134701) at 1:200. Secondary antibodies from Jackson ImmunoResearch Laboratories were Cy5 AffiniPure Donkey anti‐goat IgG (H + L; #705–175‐147), and Cy5 AffiniPure Donkey anti‐rabbit IgG (H + L; #711–175‐152). Secondary antibodies from Invitrogen were Donkey anti‐rabbit IgG (H + L) with Alexa Fluor 546 (#A10040), Donkey anti‐mouse IgG with Alexa Fluor 546 (#A10036), Donkey anti‐mouse IgG with Alexa Fluor 488 (#A21202), and Goat anti‐chicken IgY (H + L) with Alexa Fluor 488 (#A11039). Images were taken with an AMG EVOS (Life Technologies) and with a Zeiss LSM 710 confocal microscope.

### 2.5 Intracytoplasmic sperm injection (ICSI)

Metaphase II‐arrested oocytes were collected from superovulated BDF1 females (3–4 weeks) or C57BL/6J females (3–4 weeks). Cumulus cells were removed using hyaluronidase (Sigma, #H3884) at 37 °C for 5 min. The cauda epididymis was washed twice with Dulbecco's phosphate buffered saline (DPBS, Gibco, #14190094), then directly put in 1 mL of EmbryoMax Human Tubal Fluid (Merck Millipore, #MR‐070‐D). Cell suspensions were exposed to ultrasound for 1–2 min. Spermatozoa without tail were picked up into a blunt piezo‐driven pipette with a tip of 10–15 μm diameter. A single sperm head was injected into a single oocyte in a droplet of M2 medium (Sigma, #M7167) containing 5 μg/mL cytochalasin B (Sigma, #C6762) using a pipette with a tip of 10–15 μm diameter, and a piezo micromanipulator controller (Japan Prime Tech, #PMAS‐CT150). Injected oocytes were maintained in KSOM medium (Merck Millipore, #MR‐106‐D) at 37 °C with 5% CO_2_ in air. Two‐cell embryos were transferred into the oviducts of pseudopregnant ICR females. Offspring were born on day 19.5 of gestation.

## 3 RESULTS

### 3.1 Derivation of SSC lines

**Figure 1:**
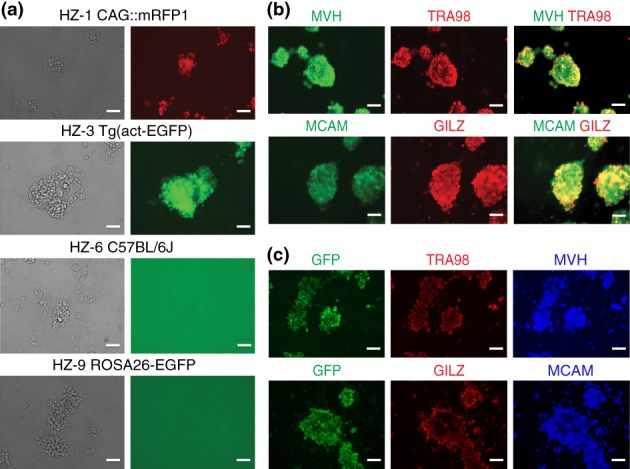
SSC lines in culture. (a) Left, brightfield images, and right, fluorescence images. Strain from which the SSC line was derived is indicated. HZ‐1 displays the red intrinsic red fluorescence of RFP. HZ‐3 displays the intrinsic green fluorescence of GFP. HZ‐6 and HZ‐9 do not display the intrinsic green fluorescence of GFP. (b) Immunofluorescence of HZ‐6 (derived from C57BL/6J) with antibodies for MVH, TRA98, MCAM, and GILZ. (c) Immunofluorescence of HZ‐11 (derived from B6‐GFP) with antibodies for GFP, TRA98, MVH, GILZ, and MCAM. Scale bars, 50 μm

We derived a dozen SSC lines from a variety of wild‐type, gene‐targeted, and transgenic mouse strains. HZ‐1 was derived from strain CAG::mRFP1 (Long et al., 2005), and displays the intrinsic red fluorescence from RFP (Figure 1a). HZ‐2 was derived from strain UBI‐EGFP (Schaefer et al., 2001). HZ‐3 was derived from strain Tg(act‐EGFP) (Okabe et al., 1997), and displays the intrinsic green fluorescence from GFP (Figure 1a). HZ‐4 was derived from strain D4/XEGFP (Hadjantonakis et al., 1998). HZ‐5 was derived from strain B6‐iDTR (Buch et al., 2005). HZ‐6, HZ‐7, and HZ‐10 were derived from wild‐type C57BL/6J (Figure 1a). HZ‐8 was derived from wild‐type CD‐1. HZ‐9 was derived from strain ROSA26‐EGFP (Giel‐Moloney et al., 2007), but does not display the intrinsic green fluorescence from GFP (Figure 1a). HZ‐11 and HZ‐12 were derived from strain B6‐GFP (Okabe et al., 1997). These SSC lines were cultured continuously for long periods of time (up to 1 year), and could be frozen and thawed successfully.

To confirm that the newly derived cell lines have the characteristics of SSCs, we performed immunofluorescence with antibodies for markers with various cellular localizations: cytoplasm, nucleus, and cell surface. The mouse vasa homologue (MVH) is expressed in the cytoplasm of germ cells; Vasa (also known as Ddx4) is an ATP‐dependent RNA helicase that is highly conserved among vertebrates and invertebrates (Castrillon, Quade, Wang, Quigley, & Crum, 2000; Gustafson & Wessel, 2010). The antigen detected by the TRA98 antibody is the *Gcna1* gene product (Carmell et al., 2016), and resides in the nucleus of testis germ cells (Inoue, Onohara, & Yokota, 2011). CD146, also called melanoma cell adhesion molecule (MCAM), is a transmembrane glycoprotein used as an SSC cell surface marker (Kanatsu‐Shinohara, Morimoto, & Shinohara, 2012). We also used antibodies against GILZ, the protein encoded by the gene (Koentgen et al., 2016). Cell lines derived from a C57BL/6J mouse (Figure 1b) and from a GFP‐expressing mouse (Figure 1c) were immunoreactive for MVH, TRA98, MCAM, and GILZ, confirming that they express classical markers for SSCs.

### 3.2 Testes of Tsc22d3‐conKO mice

**Figure 2:**
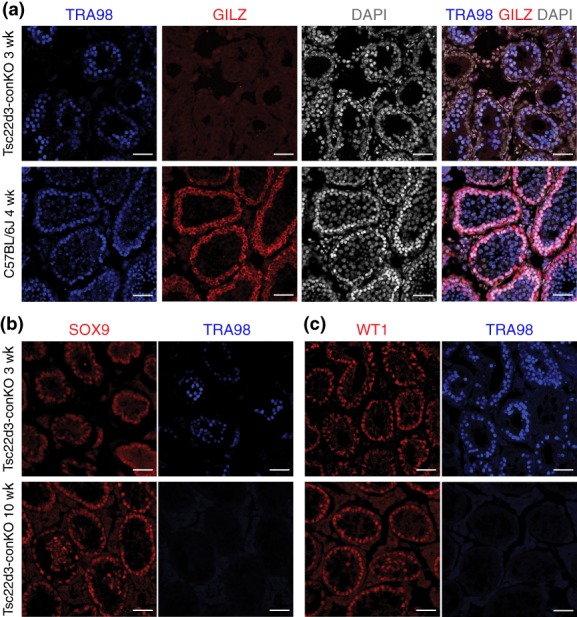
Testes of Tsc22d3‐conKO and C57BL/6J mice. (a) Immunofluorescence for TRA98 and GILZ in cryosections of testes of a Tsc22d3‐ conKO mouse at 3 weeks, and of a C57BL/6J mouse at 4 weeks. DAPI (white) serves as nuclear stain. (b) Immunofluorescence for SOX9 and TRA98 in cryosections of testes of Tsc22d3‐conKO mice at 3 weeks and 10 weeks. (c) Immunofluorescence for WT1 and TRA98 in cryosections of testes of Tsc22d3‐conKO mice at 3 weeks and 10 weeks. Scale bars, 50 μm

We performed immunofluorescence on cryosections of testes of Tsc22d3‐conKO mice and C57BL/6J mice. Immunoreactivity for GILZ is not detectable in Tsc22d3‐conKO testes at 3 weeks (Figure 2a). Immunoreactivity for GILZ colocalizes with cells expressing the germ cell marker that is detected by the TRA98 antibody (Tanaka et al., 1997) in C57BL/6J mice at 4 weeks (Figure 2a). There are TRA98+ germ cells in Tsc22d3‐conKO testes at 3 weeks but no longer at 10 weeks (Figure 2a–c). SOX9 and WT1, markers for Sertoli cells (Gao et al., 2006; Hemendinger, Gores, Blacksten, Harley, & Halberstadt, 2002; Kreidberg et al., 1993), are expressed in Tsc22d3‐conKO testes at 3 weeks and 10 weeks (Figure 2b,c).

### 3.3 Testes of Tsc22d3‐conKO mice after SSC transplantation

**Figure 3:**
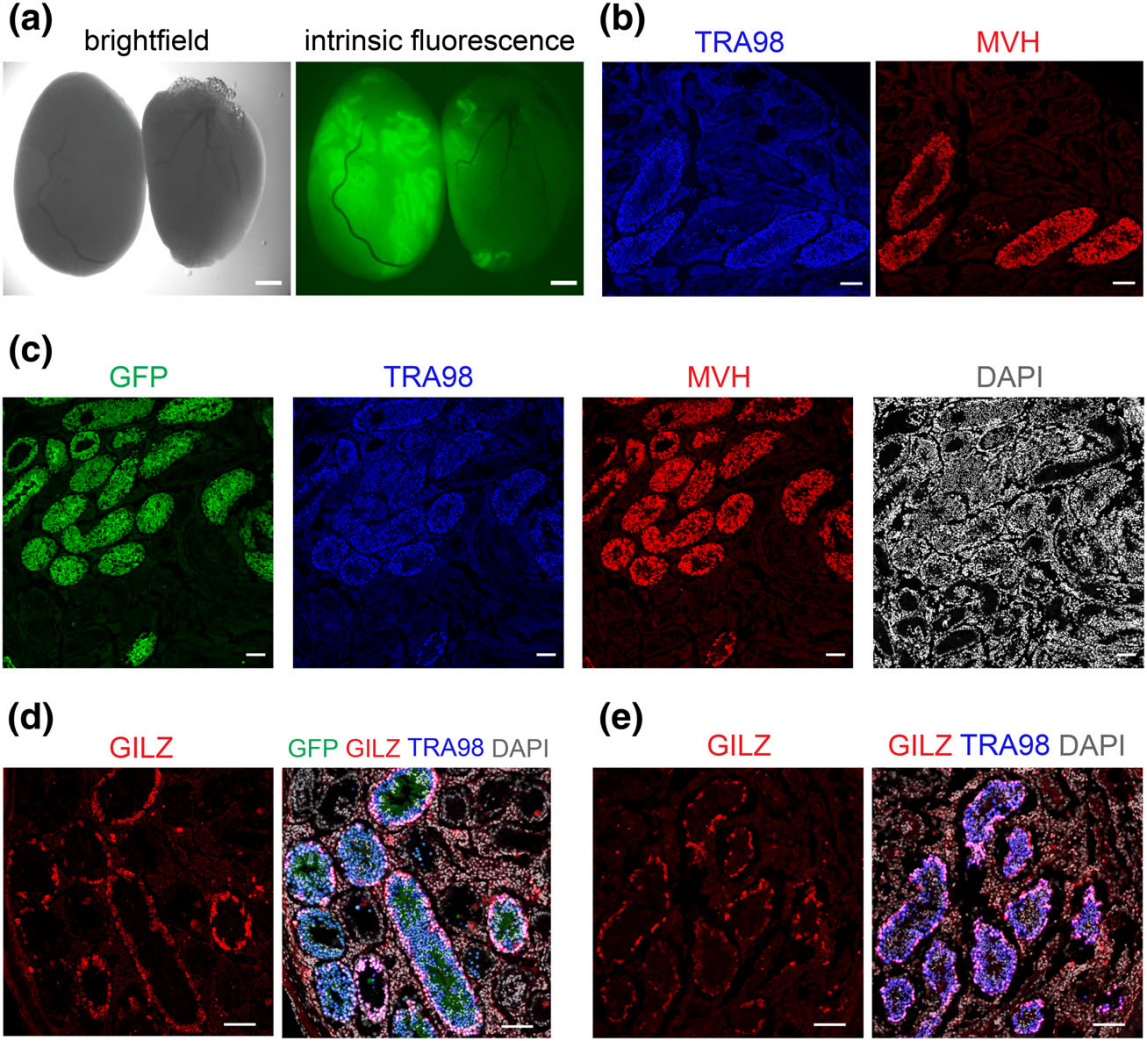
Testes of Tsc22d3‐conKO mice after SSC transplantation. (a) Macroscopic view of Tsc22d3‐conKO testes transplanted with HZ‐12 (derived from B6‐GFP). (b) Immunofluorescence for TRA98 and MVH in cryosections of a Tsc22d3‐conKO testis transplanted with HZ‐6 (derived from C57BL/6J). (c) Immunofluorescence for GFP, TRA98, and MVH in a cryosection of a Tsc22d3‐conKO testis transplanted with HZ‐11 (derived from B6‐GFP). DAPI (white) serves as nuclear stain. (d) Immunofluorescence for GILZ, TRA98, and GFP in a cryosection of a Tsc22d3‐conKO testis transplanted with HZ‐12 (derived from B6‐GFP). DAPI (white) serves as nuclear stain. (e) Immunofluorescence for GILZ and TRA98 in a cryosection of a Tsc22d3‐conKO testis transplanted with HZ‐6 (derived from C57BL/6J). DAPI (white) serves as nuclear stain. Scale bars, 2 mm in (a) and 100 μm in (b–e)

We transplanted seven SSC lines into the testes of Tsc22d3‐conKO mice (Figure 3a, Table 1), 10 mice per SSC line, for a total of 70 transplanted mice. Five SSC lines were derived from GFP+ mice: HZ‐2 (UBI‐EGFP), HZ‐3 Tg(act‐EGFP), HZ‐9 (ROSA26‐EGFP), HZ‐11, and HZ‐12 (B6‐GFP). Two SSC lines were derived from wild‐type mice: HZ‐6 and HZ‐7 (C57BL/6J). Two months after transplantation, colonization of the seminiferous tubules of Tsc22d3‐conKO testes by the GFP+ cells from HZ‐12 (derived from B6‐GFP) could be visualized readily by exposure to UV light in a macroscopic view of a testis (Figure 3a). Immunoreactivity for TRA98 and MVH was present in cryosections of a Tsc22d3‐conKO testis transplanted with HZ‐6 (derived from C57BL/6J; Figure 3b). Cryosections of a Tsc22d3‐conKO testis transplanted with HZ‐11 (derived from B6‐GFP) showed colocalization of GFP, TRA98, and MVH immunoreactivity (Figure 3c). In cryosections of a Tsc22d3‐conKO testis transplanted with HZ‐12 (derived from B6‐GFP), GILZ immunoreactivity colocalized with the GFP and TRA98 signals in seminiferous tubules (Figure 3d). Immunofluorescence in cryosections of a Tsc22d3‐conKO testis transplanted with HZ‐6 (derived from C57BL/6J) revealed numerous TRA98+ and GILZ+ cells in seminiferous tubules (Figure 3e).

**Table 1:** Transplantation of long‐term cultured SSCs

SSC line	Strain	Age of male from which SSC line was derived	Genotype	Genetic background	No. of recipients	No. of recipients analyzed	No. (%) of recipients with germline colonization
HZ‐2	UBI‐EGFP	2 weeks	Homozygous	C56BL/6 J	10	7	2 (29%)
HZ‐3	Tg(act‐EGFP)	1 week	Homozygous	C56BL/6 J	10	6	5 (83%)
HZ‐6	C57BL/6J	3 weeks	Wild‐type	C56BL/6 J	10	7	3 (43%)
HZ‐7	C57BL/6J	1 week	Wild‐type	C56BL/6 J	10	9	4 (44%)
HZ‐9	ROSA26‐EGFP	5 weeks	Heterozygous	C56BL/6 J	10	10	5 (50%)
HZ‐11	B6‐GFP	12 weeks	Hemizygous	C56BL/6 J	10	5	3 (60%)
HZ‐12	B6‐GFP	4 weeks	Hemizygous	C56BL/6 J	10	8	3 (38%)

### 3.4 Differentiation and spermatogenesis after SSC transplantation

**Figure 4:**
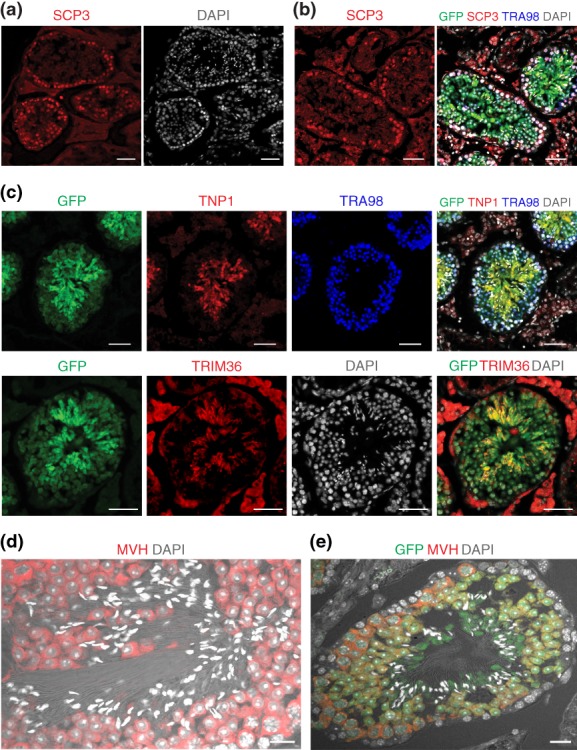
Spermatogenesis in testes of Tsc22d3‐conKO mice after SSC transplantation. (a) Immunofluorescence for SCP3 in a cryosection of a Tsc22d3‐conKO testis after transplantation of HZ‐6 (derived from C57BL/6J). DAPI (white) serves as nuclear stain. (b) Immunofluorescence for SCP3, GFP, and TRA98 in a cryosection of a Tsc22d3‐conKO testis after transplantation of HZ‐9 (derived from ROSA26‐EGFP). DAPI (white) serves as nuclear stain. (c) Immunofluorescence for GFP, TNP1, TRA98, and TRIM36 in cryosections of a Tsc22d3‐conKO testis after transplantation of HZ‐9 (derived from ROSA26‐EGFP). DAPI (white) serves as nuclear stain (d) Immunofluorescence for MVH in a cryosection of a Tsc22d3‐conKO testis after transplantation of HZ‐7 (derived from C57BL/6J). DAPI (white) serves as nuclear stain. (e) Immunofluorescence for GFP and MVH in a cryosection of a Tsc22d3‐conKO testis after transplantation of HZ‐11 (derived from B6‐GFP). DAPI (white) serves as nuclear stain. Scale bars, 50 μm in (a–c) and 20 μm in (d, e)

To determine if the transplanted SSCs are committed towards meiosis, we performed immunofluorescence for synaptonemal complex protein 3 (SCP3), a meiosis‐specific component of the axial/lateral element of the synaptonemal complex (Yuan et al., 2000). Immunofluorescence for SCP3 of Tsc22d3‐conKO testes transplanted with HZ‐6 (derived from C57BL/6J) showed numerous immunoreactive cells (Figure 4a). We detected costaining with SCP3, GFP, and TRA98 antibodies in a Tsc22d3‐conKO testis transplanted with HZ‐9 (derived from ROSA26‐EGFP; Figure 4b). Thus, the transplanted donor SSCs can proceed to meiosis after transplantation into Tsc22d3‐conKO testes. Next, we performed immunofluorescence for transition nuclear protein 1 (TNP1), a marker for elongating and condensing spermatids (Dadoune, 1995; Han et al., 2016; Yassine et al., 2015), and for RNF98 (also known as Trim36 and Haprin), a marker for elongated spermatids and mature sperm (Kitamura, Nishimura, Nishimune, & Tanaka, 2005; Kitamura, Tanaka, & Nishimune, 2003). We found that HZ‐9 reinitiated spermatogenesis in a Tsc22d3‐conKO testis on transplantation, and that cells developed into haploid cells (Figure 4c). We observed sperm with tail in seminiferous tubules of a Tsc22d3‐conKO testis after transplantation with HZ‐7 (derived from C57BL/6J; Figure 4d) and with HZ‐11 (derived from B6‐GFP; Figure 4e).

### 3.5 Germline transmission from transplanted SSCs

**Figure 5:**
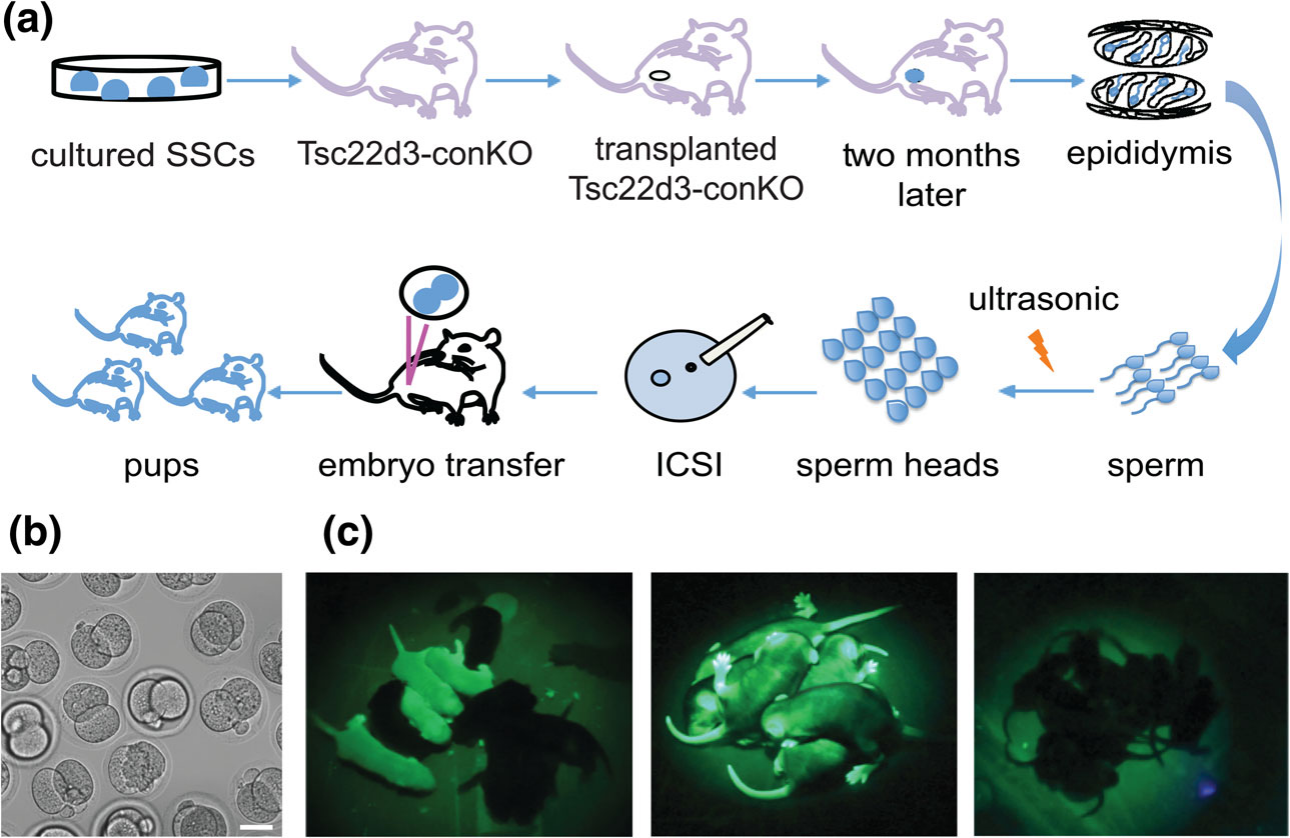
Offspring generated by ICSI with sperm from testes of Tsc22d3‐conKO mice after SSC transplantation. (a) Experimental strategy. Cultured SSCs were transplanted into the testes of Tsc22d3‐conKO mice. Two months later, epididymis spermatozoa were isolated and exposed to ultrasound, and sperm heads were injected into oocytes by ICSI. Following 2‐cell embryo transfer, live offspring were obtained. (b) Brightfield image of 2‐cell embryos after ICSI into BDF1 oocytes with sperm from a Tsc22d3‐conKO male transplanted with HZ‐9 (derived from ROSA26‐EGFP). Scale bar, 50 μm. (c) Pups exposed to UV light. Left panel, pups obtained with HZ‐9, which is hemizygous for the targeted mutation in the ROSA26 locus. Four out of 10 pups display the intrinsic green fluorescence of GFP. Middle panel, pups obtained with HZ‐3 (derived from Tg(act‐EGFP)), which is homozygous for the transgene. All pups express the intrinsic green fluorescence of GFP. Right panel, pups obtained with HZ‐6 (derived from C57BL/6J). No pups express the intrinsic green fluorescence of GFP

Males were mated with C57BL/6J females 2 months after transplantation, but no offspring was obtained after 5–14 months. We proceeded to apply the assisted reproduction technique ICSI, injecting sperm without tail into BDF1 (C57BL/6J x DBA/2) oocytes or C57BL/6J oocytes (Figure 5a). Zygotes developed into 2‐cell embryos after 12–24 hr culture in KSOM medium (Figure 5b). Following transplantation of 2‐cell embryos into oviducts of pseudopregnant ICR females, we obtained a total of 96 live pups: 79 pups using BDF1 oocytes and 17 pups using C57BL/6J oocytes (Table 2). The birth rate for ICSI using C57BL/6J oocytes is known to be very low (Sakamoto, Kaneko, & Nakagata, 2005). Pups exposed to UV light displayed the intrinsic green fluorescence of GFP in a manner that is consistent with the provenance of the SSC line from a heterozygous mouse (Figure 5c, left panel), a homozygous mouse (Figure 5c, middle panel), or a C57BL/6J mouse (Figure 5c, right panel). We confirmed the presence or absence of GFP in genomic DNA from tail biopsies with PCR (data not shown). Thus, the testicular soma of Tsc22d3‐conKO mice supports spermatogenesis and germline transmission of long‐term cultured SSC lines on transplantation.

**Table 2:** Production of offspring by ICSI

SSC line	Percentage of cultured embryos that developed to the two‐cell stage	No. of embryos transferred	No. (%) of pups	No. of GFP+ pups
B6D2F1 oocytes				
HZ‐2	77%	61	0 (0%)	0
HZ‐3	52%	102	8 (8%)	8
HZ‐6	61%	82	21 (26%)	0
HZ‐7	43%	100	22 (22%)	0
HZ‐9	50%	92	9 (10%)	4
HZ‐11	46%	100	19 (19%)	11
HZ‐12	60%	100	0 (0%)	0
C57BL/6J oocytes				
HZ‐3	80%	92	5 (5%)	5
HZ‐6	55%	54	0 (0%)	0
HZ‐7	80%	62	2 (3%)	0
HZ‐9	61%	84	0 (0%)	0
HZ‐11	65%	92	4 (4%)	1
HZ‐12	76%	80	6 (8%)	5

## 4 DISCUSSION

Spermatogonial stem cells (SSCs) continuously undergo self‐renewal to maintain the undifferentiated state, and differentiate to produce eventually spermatozoa, which transmit genetic information to the next generation (Oatley & Brinster, 2012). Recently gene expression signatures have been identified for 11 successive cell types or subtypes within the spermatogenic lineage of the mouse, starting with SSCs (Hermann et al., 2018). Transplantation of SSCs into testes can be applied to treat male infertility (Ogawa, Dobrinski, Avarbock, & Brinster, 2000); can be used to produce transgenic animals (Chapman et al., 2015; Sato et al., 2015; Wu et al., 2015); and is the most stringent functional assay to assess SSC activity. After transplantation into the seminiferous tubules, SSCs pass through the blood testis barrier that is formed by tight, adherens and gap junctions between adjacent Sertoli cells; a fraction of the transplanted SSCs migrate to the basement membrane of the seminiferous tubules; and some cells complete the process of spermatogenesis (Nagano, Avarbock, & Brinster, 1999).

Recipients for SSC transplantation have been prepared or bred in several ways over the past decades: by testicular irradiation (Withers, Hunter, Barkley, & Reid, 1974; Zhang, Shao, & Meistrich, 2006), by cooling the testes (Ehmcke, Joshi, Hergenrother, & Schlatt, 2007; Young et al., 1988; Zhang et al., 2004), by heat shock treatment (Ma et al., 2011), by experimental cryptorchidism (Mendis‐Handagama, Kerr, & de Kretser, 1990), by injection of the chemotherapeutic drug busulfan (Brinster & Avarbock, 1994; Brinster & Zimmermann, 1994; Bucci & Meistrich, 1987), and by breeding Kit^W^/Kit^Wv^ compound heterozygous mice (Brinster & Avarbock, 1994; Brinster & Zimmermann, 1994). A quarter of a century after the first reports of busulfan‐treated mice and Kit^W^/Kit^Wv^ compound heterozygous mice as recipients for spermatogonial transplantation (Brinster & Avarbock, 1994; Brinster & Zimmermann, 1994), they remain the most widely used types of recipients. But disadvantages include the morbidity and mortality (busulfan‐treated mice), and the inefficient generation of recipients by breeding (only 25% of male offspring from heterozygous parents is Kit^W^/Kit^Wv^ compound heterozygous). There is thus opportunity for improvement in identifying and optimizing a novel type of recipient that is devoid of morbidity or mortality and that can be bred efficiently in a single cross and without genotyping.

GILZ was originally discovered as an anti‐inflammatory protein that is involved in the immunosuppressive effects of glucocorticoids (D'Adamio et al., 1997). The mouse GILZ protein is encoded by the *Tsc22d3* gene, which is located on the X chromosome. Males hemizygous for a Tsc22d3 knockout were unexpectedly found to be infertile (Bruscoli et al., 2012; Ngo et al., 2013, 2013; Romero et al., 2012; Suarez et al., 2012). It is not possible to generate homozygous Tsc22d3‐KO females by breeding (Suarez et al., 2012). Hemizygous Tsc22d3‐KO males must be bred by crossing heterozygous females with wild‐type males, and 50% of the male offspring of such crosses are hemizygous and infertile. The defect in the germline appears to be intrinsic, as spermatogenesis can be restored in Tsc22d3‐KO males by transplantation of freshly prepared wild‐type germ cells (Bruscoli et al., 2012). But germline transmission of the donor haplotype, either by natural mating or with the aid of ICSI, remained to be shown for Tsc22d3‐KO mice.

Our strategy of the goGermline technology consists of mating homozygous Tsc22d3 floxed females with homozygous ROSA26‐Cre males, producing 100% male mice that are Tsc22d3‐conKO and infertile (Koentgen et al., 2016). Upon microinjection of embryonic stem cells in blastocysts or eight‐cell embryos generated in this cross, chimeras can be generated that yield 100% germline transmission of the embryonic stem‐cell derived genome (Koentgen et al., 2016). We have now evaluated Tsc22d3‐conKO males as recipients for SSC transplantation. Our newly derived SSC lines and the recipient males are in an inbred C57BL/6J background, and are thus immunologically fully compatible. We were able to generate live offspring carrying the donor haplotype from several SSC lines with the aid of ICSI.

The mechanisms of infertility of Tsc22d3‐KO mice have been studied by several groups and in several strains (Bruscoli et al., 2012; La et al., 2018; Ngo, Beaulieu, et al., 2013; Ngo, Cheng, et al., 2013; Romero et al., 2012; Suarez et al., 2012). The phenotype involves an arrest midway through the pachytene of meiosis I, massive apoptosis, SSC exhaustion, resulting in a progressive depletion of the germline, and terminating in a Sertoli cell‐only phenotype. Our contribution here is to demonstrate that the testicular soma of adult Tsc22d3‐conKO mice supports spermatogenesis and germline transmission upon transplantation of established, long‐term cultured SSC lines. The proof of principle that we have delivered paves the way for optimization of Tsc22d3‐conKO mice as a third type of recipient for SSC transplantation—a type of recipient that is devoid of morbidity and mortality and that can be bred efficiently and without genotyping. The next step is to obtain offspring by natural mating of transplanted Tsc22d3‐conKO males. We speculate that Tsc22d3‐conKO pups may be better recipients (Kubota & Brinster, 2018; Shinohara, Orwig, Avarbock, & Brinster, 2001).

## References

[dvg23295-bib-0001] Aoshima, K. , Baba, A. , Makino, Y. , & Okada, Y. (2013). Establishment of alternative culture method for spermatogonial stem cells using knockout serum replacement. PLoS One, 8, e77715 10.1371/journal.pone.0077715 24204931 PMC3810131

[dvg23295-bib-0002] Brinster, R. L. , & Avarbock, M. R. (1994). Germline transmission of donor haplotype following spermatogonial transplantation. Proceedings of the National Academy of Sciences of the United States of America, 91, 11303–11307. 7972054 10.1073/pnas.91.24.11303 PMC45219

[dvg23295-bib-0003] Brinster, R. L. , & Zimmermann, J. W. (1994). Spermatogenesis following male germ‐cell transplantation. Proceedings of the National Academy of Sciences of the United States of America, 91, 11298–11302. 7972053 10.1073/pnas.91.24.11298 PMC45218

[dvg23295-bib-0004] Bruscoli, S. , Velardi, E. , Di Sante, M. , Bereshchenko, O. , Venanzi, A. , Coppo, M. , … Riccard, C. (2012). Long glucocorticoid‐induced leucine zipper (L‐GILZ) protein interacts with ras protein pathway and contributes to spermatogenesis control. Journal of Biological Chemistry, 287, 1242–1251. 10.1074/jbc.M111.316372 22110132 PMC3256913

[dvg23295-bib-0005] Bucci, L. R. , & Meistrich, M. L. (1987). Effects of busulfan on murine spermatogenesis: Cytotoxicity, sterility, sperm abnormalities, and dominant lethal mutations. Mutation Research, 176, 259–268. 3807936 10.1016/0027-5107(87)90057-1

[dvg23295-bib-0006] Buch, T. , Heppner, F. L. , Tertilt, C. , Heinen, T. J. A. J. , Kremer, M. , Wunderlich, F. T. , … Waisman, A. (2005). A Cre‐inducible diphtheria toxin receptor mediates cell lineage ablation after toxin administration. Nature Methods, 2, 419–426. 10.1038/nmeth762 15908920

[dvg23295-bib-0007] Carmell, M. A. , Dokshin, G. A. , Skaletsky, H. , Hu, Y. C. , van Wolfswinkel, J. C. , Igarashi, K. J. , … Page, D. C. (2016). A widely employed germ cell marker is an ancient disordered protein with reproductive functions in diverse eukaryotes. eLife, 5, e19993 10.7554/eLife19993 27718356 PMC5098910

[dvg23295-bib-0008] Castrillon, D. H. , Quade, B. J. , Wang, T. Y. , Quigley, C. , & Crum, C. P. (2000). The human VASA gene is specifically expressed in the germ cell lineage. Proceedings of the National Academy of Sciences of the United States of America, 97, 9585–9590. 10.1073/pnas.160274797 10920202 PMC16908

[dvg23295-bib-0009] Chapman, K. M. , Medrano, G. A. , Jaichander, P. , Chaudhary, J. , Waits, A. E. , Nobrega, M. A. , … Hamra, F. K. (2015). Targeted germline modifications in rats using CRISPR/Cas9 and spermatogonial stem cells. Cell Reports, 10, 1828–1835. 10.1016/j.celrep.2015.02.040 25772367 PMC4376630

[dvg23295-bib-0010] D'Adamio, F. , Zollo, O. , Moraca, R. , Ayroldi, E. , Bruscoli, S. , Bartoli, A. , … Riccardi, C. (1997). A new dexamethasone‐induced gene of the leucine zipper family protects T lymphocytes from TCR/CD3‐activated cell death. Immunity, 7, 803–812. 9430225 10.1016/s1074-7613(00)80398-2

[dvg23295-bib-0011] Dadoune, J. P. (1995). The nuclear status of human sperm cells. Micron, 26, 323–345. 8574523 10.1016/0968-4328(95)00007-0

[dvg23295-bib-0012] Ehmcke, J. , Joshi, B. , Hergenrother, S. D. , & Schlatt, S. (2007). Aging does not affect spermatogenic recovery after experimentally induced injury in mice. Reproduction, 133, 75–83. 10.1530/REP-06-0148 17244734

[dvg23295-bib-0013] Ganguli, N. , Wadhwa, N. , Usmani, A. , Kunj, N. , Ganguli, N. , Sarkar, R. K. , … Majumdar, S. S. (2016). An efficient method for generating a germ cell depleted animal model for studies related to spermatogonial stem cell transplantation. Stem Cell Research & Therapy, 7(142), 142 10.1186/s13287-016-0405-1 27659063 PMC5032248

[dvg23295-bib-0014] Gao, F. , Maiti, S. , Alam, N. , Zhang, Z. , Deng, J. M. , Behringer, R. R. , … Huff, V. (2006). The Wilms tumor gene, *Wt1*, is required for *Sox9* expression and maintenance of tubular architecture in the developing testis. Proceedings of the National Academy of Sciences of the United States of America, 103, 11987–11992. 10.1073/pnas.0600994103 16877546 PMC1567685

[dvg23295-bib-0015] Giel‐Moloney, M. , Krause, D. S. , Chen, G. , Van Etten, R. A. , & Leiter, A. B. (2007). Ubiquitous and uniform in vivo fluorescence in ROSA26‐EGFP BAC transgenic mice. Genesis, 45, 83–89. 10.1002/dvg.20269 17269129 PMC2121618

[dvg23295-bib-0016] Gustafson, E. A. , & Wessel, G. M. (2010). Vasa genes: Emerging roles in the germ line and in multipotent cells. BioEssays, 32, 626–637. 10.1002/bies.201000001 20586054 PMC3090673

[dvg23295-bib-0017] Hadjantonakis, A. K. , Gertsenstein, M. , Ikawa, M. , Okabe, M. , & Nagy, A. (1998). Non‐invasive sexing of preimplantation stage mammalian embryos. Nature Genetics, 19, 220–222. 10.1038/893 9662390

[dvg23295-bib-0018] Han, H. , Wang, A. , Liu, L. , Zhao, G. , Su, J. , Wang, B. , … Li, X. (2016). Testicular characteristics and the block to spermatogenesis in mature hinny. Asian‐Australasian Journal of Animal Sciences, 29, 793–800. 10.5713/ajas.15.0670 26954128 PMC4852245

[dvg23295-bib-0019] Hemendinger, R. A. , Gores, P. , Blacksten, L. , Harley, V. , & Halberstadt, C. (2002). Identification of a specific Sertoli cell marker, Sox9, for use in transplantation. Cell Transplantation, 11, 499–505. 12428738

[dvg23295-bib-0020] Hermann, B. P. , Cheng, K. , Singh, A. , Roa‐De La Cruz, L. , Mutoji, K. N. , Chen, I. C. , … McCarrey, J. R. (2018). The mammalian spermatogenesis single‐cell transcriptome, from spermatogonial stem cells to spermatids. Cell Reports, 25, 1650–1667.e8. 10.1016/j.celrep.2018.10.026 30404016 PMC6384825

[dvg23295-bib-0021] Inoue, N. , Onohara, Y. , & Yokota, S. (2011). Expression of a testis‐specific nuclear protein, TRA98, in mouse testis during spermatogenesis. A quantitative and qualitative immunoelectron microscopy (IEM) analysis. Open Journal of Cell Biology, 1, 11–20. 10.4236/ojcb.2011.11002

[dvg23295-bib-0022] Kanatsu‐Shinohara, M. , Ikawa, M. , Takehashi, M. , Ogonuki, N. , Miki, H. , Inoue, K. , … Shinohara, T. (2006). Production of knockout mice by random or targeted mutagenesis in spermatogonial stem cells. Proceedings of the National Academy of Sciences of the United States of America, 103, 8018–8023. 10.1073/pnas.0601139103 16679411 PMC1472422

[dvg23295-bib-0023] Kanatsu‐Shinohara, M. , Morimoto, H. , & Shinohara, T. (2012). Enrichment of mouse spermatogonial stem cells by melanoma cell adhesion molecule expression. Biology of Reproduction, 87, 1–10. 10.1095/biolreprod.112.103861 23053437

[dvg23295-bib-0024] Kanatsu‐Shinohara, M. , Ogonuki, N. , Inoue, K. , Miki, H. , Ogura, A. , Toyokuni, S. , & Shinohara, T. (2003). Long‐term proliferation in culture and germline transmission of mouse male germline stem cells. Biology of Reproduction, 69, 612–616. 10.1095/biolreprod.103.017012 12700182

[dvg23295-bib-0025] Kanatsu‐Shinohara, M. , Ogonuki, N. , Inoue, K. , Ogura, A. , Toyokuni, S. , Honjo, T. , & Shinohara, T. (2002). Allogeneic offspring produced by male germ line stem cell transplantation into infertile mouse testis. Biology of Reproduction, 68, 167–173. 10.1095/biolreprod.102.008516 12493709

[dvg23295-bib-0026] Kanatsu‐Shinohara, M. , & Shinohara, T. (2013). Spermatogonial stem cell self‐renewal and development. Annual Review of Cell and Developmental Biology, 29, 163–187. 10.1146/annurev-cellbio-101512-122353 24099084

[dvg23295-bib-0027] Kanatsu‐Shinohara, M. , Toyokuni, S. , Morimoto, T. , Matsui, S. , Honjo, T. , & Shinohara, T. (2003). Functional assessment of self‐renewal activity of male germline stem cells following cytotoxic damage and serial transplantation. Biology of Reproduction, 68, 1801–1807. 10.1095/biolreprod.102.012575 12606387

[dvg23295-bib-0028] Kitamura, K. , Nishimura, H. , Nishimune, Y. , & Tanaka, H. (2005). Identification of human HAPRIN potentially involved in the acrosome reaction. Journal of Andrology, 26, 511–518. 10.2164/jandrol.04189 15955891

[dvg23295-bib-0029] Kitamura, K. , Tanaka, H. , & Nishimune, Y. (2003). Haprin, a novel haploid germ cell‐specific RING finger protein involved in the acrosome reaction. Journal of Biological Chemistry, 278, 44417–44423. 10.1074/jbc.M304306200 12917430

[dvg23295-bib-0030] Koentgen, F. , Lin, J. , Katidou, M. , Chang, I. , Khan, M. , Watts, J. , & Mombaerts, P. (2016). Exclusive transmission of the embryonic stem cell‐derived genome through the mouse germline. Genesis, 54, 326–333. 10.1002/dvg.22938 27012318 PMC5084746

[dvg23295-bib-0031] Kreidberg, J. A. , Sariola, H. , Loring, J. M. , Maeda, M. , Pelletier, J. , Housman, D. , & Jaenisch, R. (1993). WT‐1 is required for early kidney development. Cell, 74, 679–691. 8395349 10.1016/0092-8674(93)90515-r

[dvg23295-bib-0032] Kubota, H. , Avarbock, M. R. , & Brinster, R. L. (2004). Growth factors essential for self‐renewal and expansion of mouse spermatogonial stem cells. Proceedings of the National Academy of Sciences of the United States of America, 101, 16489–16494. 10.1073/pnas.0407063101 15520394 PMC534530

[dvg23295-bib-0033] Kubota, H. , & Brinster, R. L. (2018). Spermatogonial stem cells. Biology of Reproduction, 99, 52–74. 10.1093/biolre/ioy077 29617903 PMC6692861

[dvg23295-bib-0034] La, H. M. , Chan, A. L. , Legrand, J. M. D. , Rossello, F. J. , Gangemi, C. G. , Papa, A. , … Hobbs, R. M. (2018). GILZ‐dependent modulation of mTORC1 regulates spermatogonial maintenance. Development, 145, dev165324 10.1242/dev.165324 30126904

[dvg23295-bib-0035] Long, J. Z. , Lackan, C. S. , & Hadjantonakis, A. K. (2005). Genetic and spectrally distinct in vivo imaging: Embryonic stem cells and mice with widespread expression of a monomeric red fluorescent protein. BMC Biotechnology, 5, 20 10.1186/1472-6750-5-20 15996270 PMC1192791

[dvg23295-bib-0036] Ma, W. , An, L. , Wu, Z. , Wang, X. , Guo, M. , Miao, K. , … Tian, J. (2011). Efficient and safe recipient preparation for transplantation of mouse spermatogonial stem cells: Pretreating testes with heat shock. Biology of Reproduction, 85, 670–677. 10.1095/biolreprod.110.089623 21593478

[dvg23295-bib-0037] Ma, W. , Wang, J. , Gao, W. , & Jia, H. (2018). The safe recipient of SSC transplantation prepared by heat shock with busulfan treatment in mice. Cell Transplantation, 27, 1451–1458. 10.1177/0963689718794126 30187774 PMC6180719

[dvg23295-bib-0038] Mendis‐Handagama, S. M. , Kerr, J. B. , & de Kretser, D. M. (1990). Experimental cryptorchidism in the adult mouse: I. Qualitative and quantitative light microscopic morphology. Journal of Andrology, 11, 539–547. 1982285

[dvg23295-bib-0039] Nagano, M. , Avarbock, M. R. , & Brinster, R. L. (1999). Pattern and kinetics of mouse donor spermatogonial stem cell colonization in recipient testes. Biology of Reproduction, 60, 1429–1436. 10330102 10.1095/biolreprod60.6.1429 PMC5511737

[dvg23295-bib-0040] Ngo, D. , Beaulieu, E. , Gu, R. , Leaney, A. , Santos, L. , Fan, H. , … Morand, E. F. (2013). Divergent effects of endogenous and exogenous glucocorticoid‐induced leucine zipper in animal models of inflammation and arthritis. Arthritis and Rheumatism, 65, 1203–1212. 10.1002/art.37858 23335223

[dvg23295-bib-0041] Ngo, D. , Cheng, Q. , O'Connor, A. E. , DeBoer, K. D. , Lo, C. Y. , Beaulieu, E. , … Morand, E. F. (2013). Glucocorticoid‐induced leucine zipper (GILZ) regulates testicular FOXO1 activity and spermatogonial stem cell (SSC) function. PLoS One, 8, e59149 10.1371/journal.pone.0059149 23516608 PMC3597624

[dvg23295-bib-0042] Oatley, J. M. , & Brinster, R. L. (2012). The germline stem cell niche unit in mammalian testes. Physiological Reviews, 92, 577–595. 10.1152/physrev.00025.2011 22535892 PMC3970841

[dvg23295-bib-0043] Ogawa, T. , Aréchaga, J. M. , Avarbock, M. R. , & Brinster, R. L. (1997). Transplantation of testis germinal cells into mouse seminiferous tubules. The International Journal of Developmental Biology, 41, 111–122. 9074943

[dvg23295-bib-0044] Ogawa, T. , Dobrinski, I. , Avarbock, M. R. , & Brinster, R. L. (1999). Xenogeneic spermatogenesis following transplantation of hamster germ cells to mouse testes. Biology of Reproduction, 60, 515–521. 9916022 10.1095/biolreprod60.2.515

[dvg23295-bib-0045] Ogawa, T. , Dobrinski, I. , Avarbock, M. R. , & Brinster, R. L. (2000). Transplantation of male germ line stem cells restores fertility in infertile mice. Nature Medicine, 6, 29–34. 10.1038/71496 PMC4879876 10613820

[dvg23295-bib-0046] Okabe, M. , Ikawa, M. , Kominami, K. , Nakanishi, T. , & Nishimune, Y. (1997). “Green mice” as a source of ubiquitous green cell. FEBS Letters, 407, 313–319. 9175875 10.1016/s0014-5793(97)00313-x

[dvg23295-bib-0047] Qin, Y. , Liu, L. , He, Y. , Ma, W. , Zhu, H. , Liang, M. , … Wang, D. (2016). Testicular injection of busulfan for recipient preparation in transplantation of spermatogonial stem cells in mice. Reproduction, Fertility and Development, 28, 1916–1925. 10.1071/RD14290 26111862

[dvg23295-bib-0048] Romero, Y. , Vuandaba, M. , Suarez, P. , Grey, C. , Calvel, P. , Conne, B. , … Nef, S. (2012). The glucocorticoid‐induced leucine zipper (GILZ) is essential for spermatogonial survival and spermatogenesis. Sexual Development, 6, 169–177. 10.1159/000338415 22571926 PMC6187839

[dvg23295-bib-0049] Russell, W. M. S. , & Burch, R. L. (1959). The principles of humane experimental technique. London: Methuen.

[dvg23295-bib-0050] Sakamoto, W. , Kaneko, T. , & Nakagata, N. (2005). Use of frozen‐thawed oocytes for efficient production of normal offspring from cryopreserved mouse spermatozoa showing low fertility. Comparative Medicine, 55, 136–139. 15884774

[dvg23295-bib-0051] Sato, T. , Sakuma, T. , Yokonishi, T. , Katagiri, K. , Kamimura, S. , Ogonuki, N. , … Ogawa, T. (2015). Genome editing in mouse spermatogonial stem cell lines using TALEN and double‐nicking CRISPR/Cas9. Stem Cell Reports, 5, 75–82. 10.1016/j.stemcr.2015.05.011 26095606 PMC4618438

[dvg23295-bib-0052] Schaefer, B. C. , Schaefer, M. L. , Kappler, J. W. , Marrack, P. , & Kedl, R. M. (2001). Observation of antigen‐dependent CD8+ T‐cell/dendritic cell interactions in vivo. Cellular Immunology, 214, 110–122. 10.1006/cimm.2001.1895 12088410

[dvg23295-bib-0054] Shinohara, T. , Orwig, K. E. , Avarbock, M. R. , & Brinster, R. L. (2001). Remodeling of the postnatal mouse testis is accompanied by dramatic changes in stem cell number and niche accessibility. Proceedings of the National Academy of Sciences of the United States of America, 98, 6186–6191. 11371640 10.1073/pnas.111158198 PMC33443

[dvg23295-bib-0055] Suarez, P. E. , Rodriguez, E. G. , Soundararajan, R. , Mérillat, A. M. , Stehle, J. C. , Rotman, S. , … Hummler, E. (2012). The glucocorticoid‐induced leucine zipper (Gilz/Tsc22d3‐2) gene locus plays a crucial role in male fertility. Molecular Endocrinology, 26, 1000–1013. 10.1210/me.2011-1249 22556341 PMC5416992

[dvg23295-bib-0056] Tanaka, H. , Pereira, L. A. , Nozaki, M. , Tsuchida, J. , Sawada, K. , Mori, H. , & Nishimune, Y. (1997). A germ cell‐specific nuclear antigen recognized by a monoclonal antibody raised against mouse testicular germ cells. International Journal of Andrology, 20, 361–366. 9568529 10.1046/j.1365-2605.1998.00080.x

[dvg23295-bib-0057] Wang, D. Z. , Zhou, X. H. , Yuan, Y. L. , & Zheng, X. M. (2010). Optimal dose of busulfan for depleting testicular germ cells of recipient mice before spermatogonial transplantation. Asian Journal of Andrology, 12, 263–270. 10.1038/aja.2009.67 20010847 PMC3739084

[dvg23295-bib-0058] Withers, H. R. , Hunter, N. , Barkley, H. T. , & Reid, B. O. (1974). Radiation survival and regeneration characteristics of spermatogenic stem cells of mouse testis. Radiation Research, 57, 88–103. 10874929

[dvg23295-bib-0059] Wu, Y. , Zhou, H. , Fan, X. , Zhang, Y. , Zhang, M. , Wang, Y. , … Li, J. (2015). Correction of a genetic disease by CRISPR‐Cas9‐mediated gene editing in mouse spermatogonial stem cells. Cell Research, 25, 67–79. 10.1038/cr.2014.160 25475058 PMC4650588

[dvg23295-bib-0060] Yassine, S. , Escoffier, J. , Martinez, G. , Coutton, C. , Karaouzène, T. , Zouari, R. , … Arnoult, C. (2015). Dpy19l2‐deficient globozoospermic sperm display altered genome packaging and DNA damage that compromises the initiation of embryo development. MHR: Basic Science of Reproductive Medicine, 21, 169–185. 10.1093/molehr/gau099 25354700 PMC4311149

[dvg23295-bib-0061] Young, G. P. , Goldstein, M. , Phillips, D. M. , Sundaram, K. , Gunsalus, G. L. , & Bardin, C. W. (1988). Sertoli cell‐only syndrome produced by cold testicular ischemia. Endocrinology, 122, 1074–1082. 10.1210/endo-122-3-1074 3125037

[dvg23295-bib-0062] Yuan, L. , Liu, J. G. , Zhao, J. , Brundell, E. , Daneholt, B. , & Höög, C. (2000). The murine SCP3 gene is required for synaptonemal complex assembly, chromosome synapsis, and male fertility. Molecular Cell, 5, 73–83. 10678170 10.1016/s1097-2765(00)80404-9

[dvg23295-bib-0063] Zhang, M. , Zhou, H. , Zheng, C. , Xiao, J. , Zuo, E. , Liu, W. , … Li, J. (2014). The roles of testicular c‐kit positive cells in de novo morphogenesis of testis. Scientific Reports, 4(5936). 10.1038/srep05936 PMC4119999 25088917

[dvg23295-bib-0064] Zhang, Z. , Shao, S. , & Meistrich, M. L. (2006). Irradiated mouse testes efficiently support spermatogenesis derived from donor germ cells of mice and rats. Journal of Andrology, 27, 365–375. 10.2164/jandrol.05179 16339450

[dvg23295-bib-0065] Zhang, Z. , Short, R. V. , Meehan, T. , de Kretser, D. M. , Renfree, M. B. , & Loveland, K. L. (2004). Functional analysis of the cooled rat testis. Journal of Andrology, 25, 57–68. 14662787 10.1002/j.1939-4640.2004.tb02759.x

